# Fractal Theory and the Estimation of Extreme Floods

**DOI:** 10.6028/jres.099.036

**Published:** 1994

**Authors:** Donald L. Turcotte

**Affiliations:** Department of Geological Sciences, Cornell University, Ithaca, NY 14853

**Keywords:** Brownian walks, floods, fractals, Gaussian noises, time series

## Abstract

Floods and draughts constitute extreme values of great consequence to society. A wide variety of statistical techniques have been applied to the evaluation of the flood hazard. A primary difficulty is the relatively short time span over which historical data are available, and quantitative estimates for palcofloods are generally suspect. It was in the context of floods that Hurst introduced the concept of the rescaled range. This was subsequently extended by Mandelbrot and his colleagues to concepts of fractional Gaussian noises and fractional Brownian walks. These studies introduced the controversial possibility that the extremes of floods and droughts could be fractal. An extensive study of flood gauge records at 1200 stations in the United States indicates a good correlation with fractal statistics. It is convenient to introduce the parameter *F* which is the ratio of the 10 year flood to the 1-year flood; for fractal statistics *F* is also the ratio of the 100 year flood to the 10 year flood and the ratio of the 1000 year flood to the 100 year flood. It is found that the parameter *F* has strong regional variations associated with climale. The acceptance of power-law statistics rather than exponentially based statistics would lead to a far more conservative estimate of future flood hazards.

## 1. Introduction

The flow in a river can generally be considered a time series. The extreme values in the time series constitute floods. Floods present a severe natural hazard; in order to assess the hazard and to allocate resources for its mitigation it is necessary to make flood-frequency hazard assessments. The integral of the flow in a river is required for the design of reservoirs and to assess available water supplies during periods of drought.

One estimate of the severity of a flood is the peak discharge at a station *V*. The magnitude of the peak discharge is affected by a variety of circumstances including: (1) The amount of rainfall produced by the storm or storms in question, (2) the upstream drainage area, (3) the saturation of the soil in the drainage area, (4) the topography, soil type, and vegetation in the drainage area, and (5) whether snow melt is involved. In addition dams, stream channelization, and other man-made modifications can affect the severity of floods.

In order to estimate the severity of future floods, historical records are used to provide flood-frequency estimates. Unfortunately, this record generally covers a relatively short time span and no general basis has been accepted for its extrapolation. Quantitative estimates of peak discharges associated with paleofloods are generally not sufficiently accurate to be of much value. A wide variety of geostatistical distributions have been applied to flood-frequency forecasts, often with quite divergent predictions. Examples of distributions used include power law (fractal), log normal, gamma, Gumbel, log Gumbel, Hazen, and log Pearson. Many discussions of this work appear in the literature [1–7].

An independent approach to reservoir storage was developed by Hurst [8, 9]. Hurst spent his life studying the flow characteristics of the Nile and introduced the rescaled range (*R/S*) analysis. He found that the variations of the storage (the range) scaled with the time period considered as a power law. Mandelbrot and Wallis [10–13] introduced the concepts of fractional Gaussian noises and fractional Brownian walks and related these to *R/S* analysis; all are recognized as fractal distributions. They also introduced the Noah and Joseph effects. The Noah affect is the skewness of the distribution of flows in a river and the Joseph effect is the persistence of the flows. Although the concepts introduced by Hurst and Mandelbrot and Wallis have been considered in a wide variety of applications [14], they have not influenced approaches to flood-frequency forecasting. This point will be a central feature of this paper along with a general discussion of the applicability of fractal statistics.

## 2. Analysis

In most cases the flow in a river is a continuous function of time, thus it is appropriate to treat the flow as a time series. It is straightforward to study the spectral characteristics of the time series by determining the coefficients of a Fourier expansion. For most river flows there will be a strong annual peak associated with seasonal variations in rainfall. However, it is of interest to examine the longer range trends in the data. If the Fourier coefficients have a power-law dependence on frequency over a significant range of frequencies a fractal dependence is obtained (with some constraints on the power).

If $\dot{V}$ (*t*) is the volumetric flow in a river as a function of time, the condition that the flow is fractal requires that

$$\mathrm{Pr}\left[\frac{\dot{V}\left(t+T\right)-\dot{V}\left(t\right)}{T^{II}}\leq y\right]=f\left(y\right),$$ (1)

where $\dot{V}\left(t+T\right)-\dot{V}\left(t\right)$ is the difference in flow after a time *T*, *H* is known as the Hausdorff measure, and *f*(*y*) is a normalized cumulative probability distribution function. When *f*(*y*) is the error function and *H* = 1/2 this relation defines a Brownian walk. If 0 < H < 1 and *f*(*y*) is the error function, this relation defines fractional Brownian walks. The fractal dimension of a fractional Brownian walk is related to the Hausdorff measure by [15]

D=2−H (2)

and with 0 < *H* < 1 we have 1 < *D* < 2.

An extension of the self-similar analysis of rivers as a time series is to treat floods as a discrete fractal set. In order to avoid difficulties with annual variability we hypothesize that the peak annual discharge ${\dot{V}}_{m}$ in a time interval *T* is related to the interval by

$${\dot{V}}_{m}\left(T\right)=C_{1}T^{H}$$ (3)

with *T* an integer number of years. Self-similar river flows imply a power-law scaling of peak annual discharges and recurrence intervals.

This scale invariant distribution can also be expressed in terms of the ratio *F* of the peak discharge over a 10 year interval to the peak discharge over a 1 year interval. With self-similarity the parameter *F* is then also the ratio of the 100 year peak discharge to the 10 year peak discharge. In terms of *H* and *D* we have

$$F=10^{H}=10^{2-D}.$$ (4)

The parameter *F* is a measure of the severity of great floods.

An alternative way of writing Eq, (3) is

$$\dot{N}=C_{2}{\dot{V}}^{-\alpha },$$ (5)

where $\dot{N}$ is the number of floods per unit time with flows that exceed $\dot{V}$. This relation will be used to analyse actual flood-frequency data. The quantities $\dot{N}$ in Eq. (5) and *T* in Eq. (3) are related by

$$\dot{N}=\frac{1}{T’}$$ (6)

so that we have

$$H=\frac{1}{\alpha }$$ (7)

and from Eq. (2) we have

$$D=2-\frac{1}{\alpha }.$$ (8)

Data will be used to obtain *α*; *F*, *H*, and *D* will then be found from Eqs. (4), (7), and (8).

**Fig. 1:**
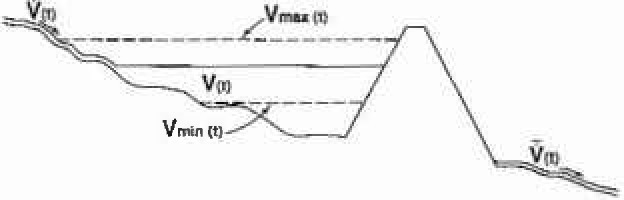
Illustration of how rescaled range (*R/S*) analysis is carried out. The flow into a reservoir is $\dot{V}\left(t\right)$ and the flow out is $\bar{\dot{V}}\left(T\right)$. The maximum volume of water in the reservoir during the period *T* is *V*_max_(*T*) and the minimum *V*_max_(*T*); the difference is the range *R*(*T*) = *V*_max_(*T*) − *V*_max_(*T*).

Before considering actual examples we will also introduce rescaled range (*R/S*) analysis. Hurst [8, 9] proposed this empirical approach to the statistics of floods and draughts. The method is illustrated in Fig. 1. Consider a reservoir behind a dam that never overflows or empties, the flow into the reservoir is $\dot{V}\left(t\right)$ and the flow out of the reservoir is $\bar{\dot{V}}\left(T\right)$ defined by

$$\bar{\dot{V}}=\frac{1}{T}\int_{0}^{T}\dot{V}\left(t\right)dt.$$ (9)

The volume of water in the reservoir *V*(*t*) is given by

$$V\left(t\right)=V\left(0\right)+\int_{0}^{T}\dot{V}\left(t\prime \right)dt\prime -t\bar{\dot{V}}\left(T\right)$$ (10)

and the range is defined by

$$R\left(T\right)=V_{\max}-V_{\min},$$ (11)

where *V*_max_ is the maximum volume and *V*_min_ the minimum volume stored during the interval *T*. The rescaled range is defined as *R/S* where *S* is the standard deviation of the flow during the period *T*

$$S\left(T\right)={\left[\frac{1}{T}\int_{0}^{T}\left(\dot{V}\right)\left(t\right)-\bar{\dot{V}}{)}^{2}\right]}^{1/2}dr.$$ (12)

Hurst et al. [16] found that for many time series the rescaled range satisfies the empirical relation

$$\frac{R}{S}={\left(\frac{T}{2}\right)}^{H_{1}}$$ (13)

where *H*_1_ is known as the Hurst exponent. Examples included river discharges, rainfall, varves, temperatures, sunspot numbers, and tree rings. In many cases the value of the Hurst exponent is near 0.7.

If a Gaussian white noise sequence of numbers is integrated or summed the result is a Brownian walk. An *R/S* analysis of the white noise sequence gives a Hurst exponent *H*_1_, thus the Hurst exponent is equal to the Hausdorff measure of the integrated signal, a Brownian walk with *H* = 0.5. Mandelbrot and Wallis [10–13] introduced the concept of fractional Gaussian noises and their integrals, fractional Brownian walks. They showed that the Hurst exponent *H*_1_ of a fractional Gaussian noise is equal to the Hausdorff measure of the corresponding fractional Brownian walk.

If 0.5 < *H*_1_ < 1 the original time series is said to be persistence; adjacent values are more strongly correlated than if they were random. The higher the value of *H*_1_, the greater the persistence. If 0 < *H*_1_ < 0.5 the original time series is said to be antipersistent; adjacent values are less correlated than if they were random.

## 3. Examples

**Fig. 2:**
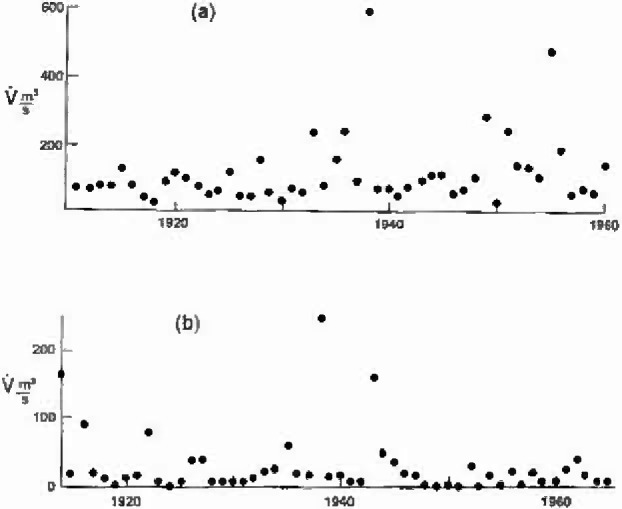
Maximum annual floods for (a) station 1–1805 on the Middle Branch of the Westfield River, Goss Heights, Massachusetts and (b) station 11-0980 in the Arroyo Seco near Pasadena, California.

We now turn to the analysis of flood-frequency records. As our first example, the 10 benchmark stations considered by Benson [2] will be studied. Benson [2] applied a variety of geostatistical distributions to the data from these stations, these will be compared with the fractal approach discussed above. The maximum annual floods for two stations are given in Fig. 2. Values for station 1–1805 on the Middle Branch of the Westfield River in Goss Heights, Massachusetts are given in Fig. 2a for the period 1911–1960 [17] and values for station 11-0980 in the Arroyo Seco near Pasadena, California are given in Fig. 2b for the period 1914–1965 [18].

In order to assess the applicability of fractal statistics the number of annual floods *N* with a peak discharge greater than $\dot{V}\left(m^{3}/s\right)$ is divided by the sampling period to give the mean number of floods per year $\dot{N}$ with a peak discharge greater than the specified value. The log $\dot{N}\left(\dot{V}\right)$ is then plotted against log $\dot{V}$. Results for station 1-1805 are given in Fig. 3a, the solid line is the least square fit of Eq. (5) with the data over the range 50 < *V* < 200 m^3^/s; large floods are omitted from the fit because of their small number. The solid line corresponds to *α* =2.3; from Eqs. (4), (7), and (8) we have *H* = 0.435, *F* =2.72, and *D* = 1.56. Results for station 11-0980 are given in Fig. 3b, the solid line is the best fit of Eq. (5) with the data over the range $10<\dot{V}<100m^{3}/s$. The solid line corresponds to *α* = 1.1; from Eqs. (4), (7), and (8) we have *H* = 0.909, *F* = 8.11, and *D* = 1.09. In both cases the fit to the scale-invariant (fractal) relation is quite good. The values of *H* and *F* in California are considerably larger than in Massachusetts. Large floods are relatively more probable in the arid climate than in the temperate climate.

The values of *H*, *D*, and *F* are given for all ten benchmark stations in Table 1. The correlations with the fractal relation (5) in Fig. 3 are typical of the ten stations. The parameter *F* is a measure of the relative severity of flooding. The higher the value of *F* the more likely that severe floods will occur. Our results show that there are clear regional trends in values of *F*. The values in the southwest including Nevada (*F* = 4.13) and New Mexico (*F* = 4.27) as well as California (*F* = 8.11) are systematically high. The high values can be attributed to the arid conditions and the rare tropical (monsoonal) storm that causes severe flooding. Central Texas (*F* = 5.24) is also high and Georgia (*F* = 3.47) is intermediate. These areas are influenced by hurricanes. The northern tier of states including Massachusetts (*F* = 2.72), Minnesota (*F* = 2.95), Nebraska (*F* = 3.47), and Wyoming (*F* = 3.31) range from low values in the east to intermediate values in the west. Washington (*F* = 2.04) has the lowest value of the stations considered; this low value is consistent with the maritime climate where extremes of climate are rare.

We have also determined the Hurst exponent for the ten benchmark stations. Values of *R/S* for *T* = 5, 10, 25, and 50 years (*R/S* = 1 for *T* = 2 by definition) are given in Fig. 4a for station 1–1805 (Westfield, MA) and in Fig. 4b for station 11–0980 (Pasadena, CA). Good correlations are obtained with (13) taking *H*_1_ = 0.67 for station 1–1805 and *H*_1_ = 0.68 for station 11-0980. Values of *H*_1_ for all ten stations are given in Table 1. The values are nearly constant with a range from 0.66 to 0.73 indicating moderate persistence. It is not surprising that the values of the Hausdorff measures *H* differ from the values of the Hurst exponent *H*_1_ since the former refers to the statistics of the flood events and the latter to the statistics of the running sum.

However, the results indicate that there is considerable variation of *α* (*H*, *D*, and *F*) but very little variation in *H*_1_. A simple explanation is that the former is sensitive to the Noah effect while the latter is sensitive to the Joseph effect. The relative scaling of floods is sensitive to the skewness of the statistical distribution but is not sensitive to the persistence of flows or floods. An important conclusion is that *R/S* analysis is not relevant to flood-frequency hazard assessments.

Many statistical distributions have been applied to historical records of floods. Benson [2] has given six statistical correlations for each of his ten benchmark stations. His results for the 2-parameter gamma (Ga), Gumbel (Gu), log Gumbel (LGu), log normal (LN), Hazen (H), and log Pearson type III (LP) are given in Fig. 5a for station 1-1805 and in Fig. 5b for station 11-0980. Also included in each figure is the self-similar (fractal) estimate (*F*). For large floods the fractal prediction (*F*) correlates best with the log Gumbel (LGu) while the other statistical techniques predict longer recurrence time for very serious floods. The fractal and log Gumbel are essentially power-law correlations whereas the others are essentially exponential.

While the ten benchmark stations provide a basis for comparing statistical approaches, they hardly made a convincing case that fractal statistics are preferable to alternatives. A principal difficulty is the relatively short time span over which reliable records have been collected. In order to try to overcome this difficulty we have analysed a large number of records and superimposed the results. We have utilized a digitized 40 year data set for 1009 stations unaffected by flood control projects [19]. The distribution of the stations over the United States is given in Fig. 6a. We will separately consider the data from the 18 hydrologie districts, these are illustrated in Fig. 6b.

The largest floods in each of the 40 water years are ordered, the largest annual flood is assigned a period of 40 years, the 2nd largest annual flood a period of 20 years, the 3rd largest annual flood a period of 13.3 years, and so forth. The log of the peak discharge for each flood is plotted against the log of its assigned period and the best straight line, i.e., from Eq. (3), is obtained. Two randomly selected examples are given in Fig. 7.

Results for station 1-860 on the Warner River in Davisville, NH, are given in Fig. 7a. The best fit straight line gives *H* = 0.68; from Eqs. (2), (4), and (7) we have *F* = 4.8, *D* = 1.32 and *α* = 1.46. Results for station 3-2305 on the Big Darby Creek in Dar-byville, OH are given in Fig. 7b. The best fit straight line gives *H* = 0.386; from Eqs. (2), (4), and (7) we have *F* = 2.43, *D* = 1.61, and *α* = 2.59.

In order to determine the quality of the fit of the data to the fractal relation Eq. (3), the ratio of the measured peak flow to the value predicted by the fractal fit is given for periods of 1, 2, 5, 10, 20, and 40 years in Fig. 8. The 111 stations from hydrologic region 3 are given in Fig. 8a, the 57 stations from region 4 in Fig. 8b, the 10 stations from region 16 in Fig. 8c, and the 100 stations from region 17 in Fig. 8d. If all points were unity the fit would be perfect. The mean deviations from the fractal relation are only a few per cent. The deviations for larger values of the period are greater as would be expected since the individual points are only a few floods. However, the mean values of the 40 year floods are close to the fractal extrapolation. This agreement provides support for the applicability of fractal statistics to the estimation of the flood hazard.

In Fig. 9a the 111 fractal fits for hydrologic region 3 are given, the fits for regions 4, 16, and 17 are given in Figs. 9b, 9c, and 9d. The peak flow at a period of 10 years was normalized by the drainage area upstream of the station. If peak flows were simply proportional to upstream drainage areas in a hydrologic district then all the plots should fall on a single band. In fact, there is more than an order of magnitude variation. This is not surprising but the details of the variations should be helpful in providing a better understanding of the flood hazard.

The regional variations in *F* are clearly illustrated in Table 2. The highest values of *F* are generally associated with the arid southwestern states in regions 12, 13, 15, and 18, the mean value of *F* for these regions is *F* = 5.03. The lowest mean value for *F* is in region 17, the Pacific Northwest, with *F* = 2.08. In some cases the standard deviations for *F* in a district are large. For district 18 (primarily California) the mean is 5.34 and the standard deviation is 2.4. In this case much of the deviation can be identified with the presence or absence of snow run off. Those stations with large upstream snow packs have relatively small values for *F* compared with those stations with little or no upstream snow packs.

## 4. Conclusions

Historical flood-frequency records have been examined to determine whether fractal (power-law) statistics are applicable. Although it must be recognized that the relatively short duration of historical records restricts the validity of conclusions; nevertheless, quite good agreement is obtained between fractal statistics and observations for 10 benchmark stations and for 1200 other stations in the United States. The basic question in terms of flood hazard assessment is whether extreme floods decay exponentially in time or as a power law. If the power-law behavior is applicable then the likelihood of severe floods is much higher and more conservative designs for dams and land use restrictions are indicated.

For fractal behavior the ratio of the 10 year to the 1 year flood F is also the ratio of the 100 year to the 10 year flood and the ratio of the 1000 year flood to the 100 year flood. We find large regional variations in values of *F*. In arid regions such as the southwestern United States the values of *F* are nearly three times the values in more temperate regions such as the northwestern and northeastern corners of the country. Smaller values of *F* are also found if upstream drainage areas have large snow packs.

The relevance of *R/S* analysis to flood frequency forecasting has also been addressed. For the ten bench mark stations we find the Hurst exponent to be *H*_1_ = 0.7 ± 0.03. This value indicates moderate persistence for the floods but also shows that determinations of Hurst exponents are not useful for flood hazard assessments. The Hurst exponent does not correlate with the fractal flood parameter *F*. In the terms introduced by Mandelbrot and Wallis [10] the Hurst exponent is sensitive to the Joseph effect or persistence of events whereas the fractal flood parameter *F* is sensitive to the Noah effect or skew-ness of the statistical distributions of floods.

It certainly remains to be demonstrated that fractal flood frequency statistics are generally valid. However, the success indicated in the results given here raises the interesting question whether the underlying physical processes are inherently fractal. Fractal statistics will be applicable to any scale invariant process. They are also applicable to dynamical systems that exhibit self-organized criticality [20]. One speculative conclusion is that the storms that generate floods are associated with the self-organized critical behavior of the atmosphere.

## Figures and Tables

**Fig. 3a:**
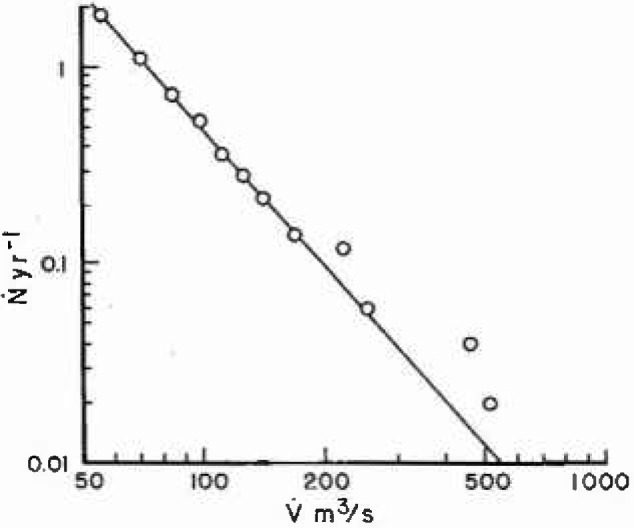
Number of floods per year with a peak discharge greater than $\dot{V}$. Station 1-1805 in Goss Heights, Massachusetts during the period 1911–1960.

**Fig. 3b:**
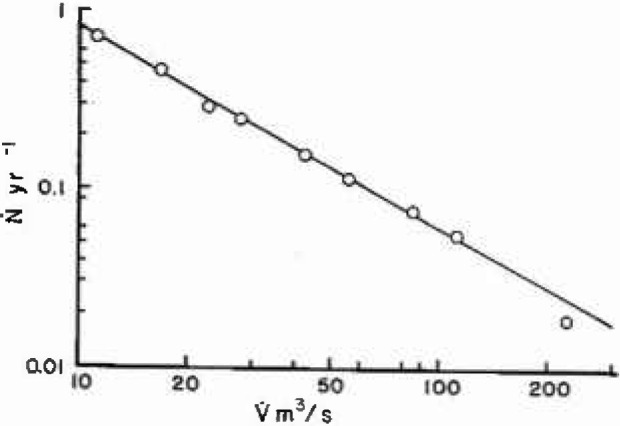
Number of floods per year with a peak discharge greater than $\dot{V}$. Station 11-0980 near Pasadena, California during the period 1914–1965.

**Fig. 4a:**
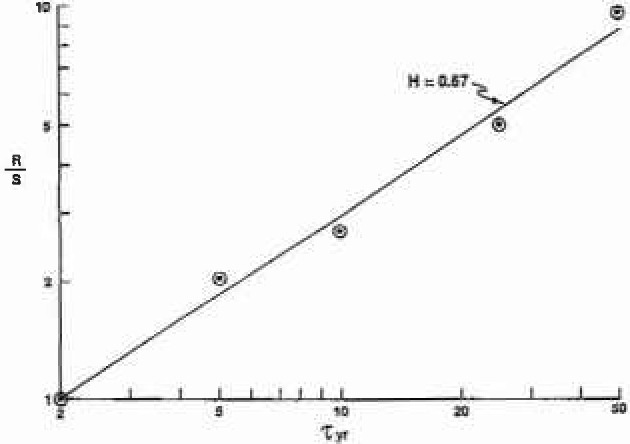
The rescaled range (*R/S*) for several inlervals *T*. Station 1–1805. The correlations are with Eq. (13) and the Hurst exponents *H*_1_ are given.

**Fig. 4b:**
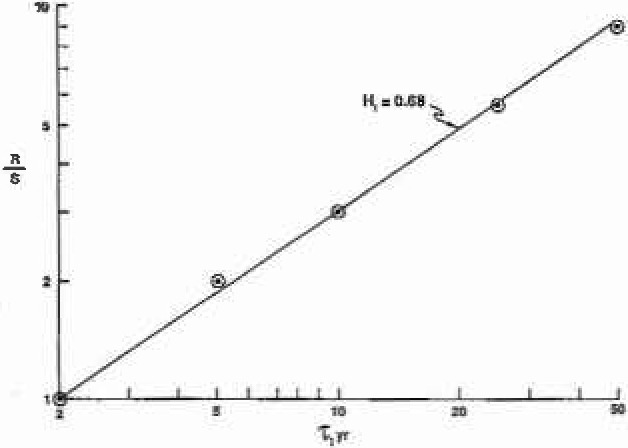
The rescaled range (*R/S*) for several intervals *T*. Station 11–0980. The correlations are with Eq. (13) and the Hursl exponcols *H*_1_ are given.

**Fig. 5a:**
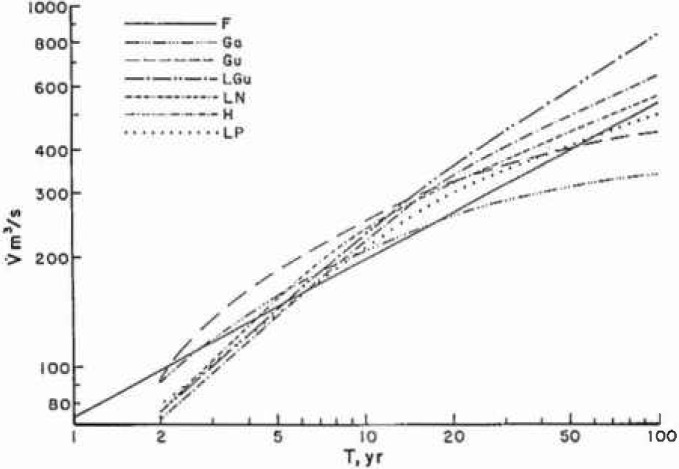
Flood frequency predictions for Station 1-1805. The peak discharge $\dot{V}$ is given as a function of recurrence intervals *T*. The scale-invariant (fractal) prediction, *F*, is compared with the six statistical predictions given by Benson (1968); 2 parameter gamma (Ga), Gumbel (Gu), log Gumbel (LGu), log normal (LN), Hazen (H), and log Pearson type III (LP).

**Fig. 5b:**
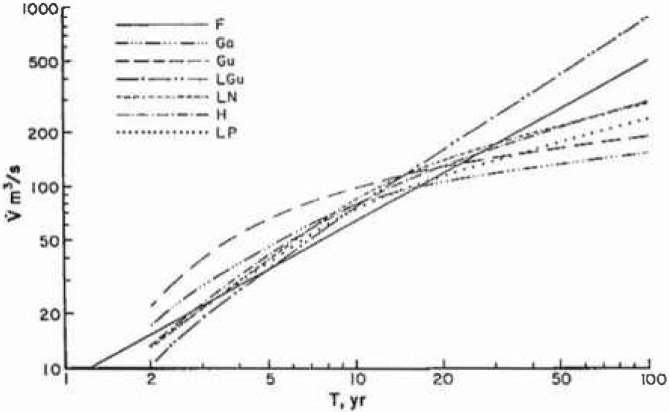
Flood frequency predictions for Station 11-0980. The peak discharge $\dot{V}$ is given as a function of recurrence intervals *T*. The scale-invariant (fractal) prediction, *F*, is compared with the six statistical predictions given by Benson (1968); 2 parameter gamma (Ga), Gumbel (Gu), log Gumbel (LGu), log normal (LN), Hazen (H), and log Pearson type III (LP).

**Fig. 6a:**
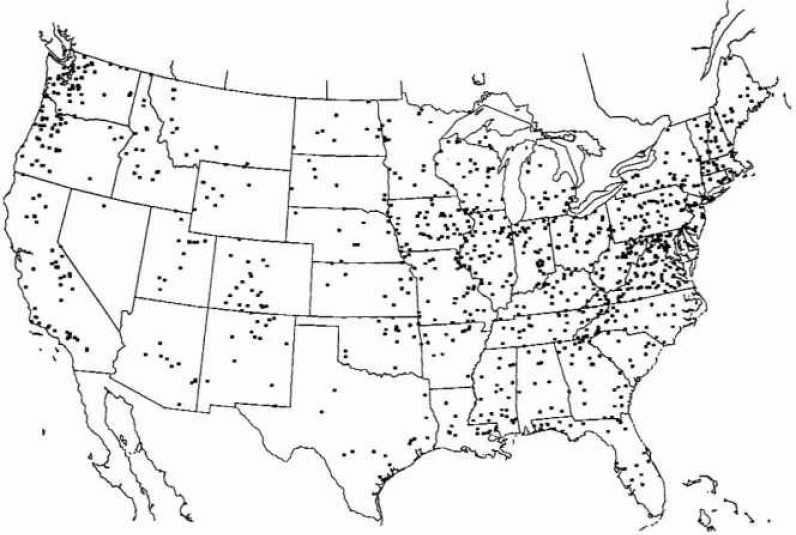
Distribution of the 1009 stations that have been analysed.

**Fig. 6b:**
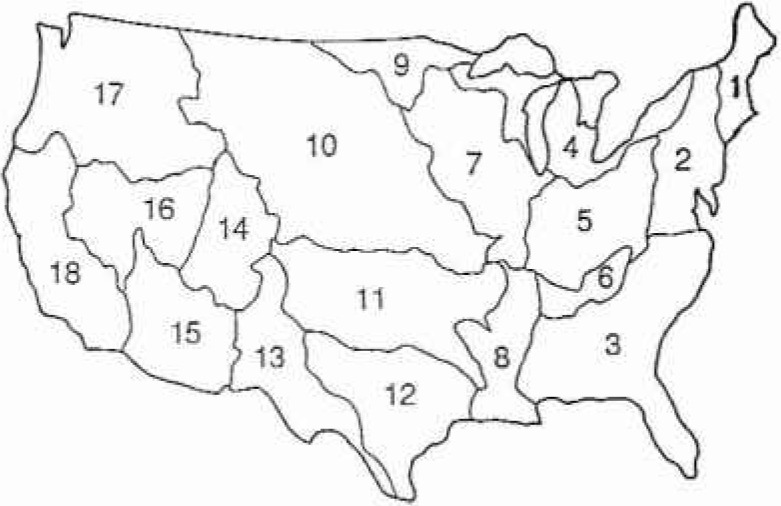
Hydrologic regions of the continental United States.

**Fig. 7a:**
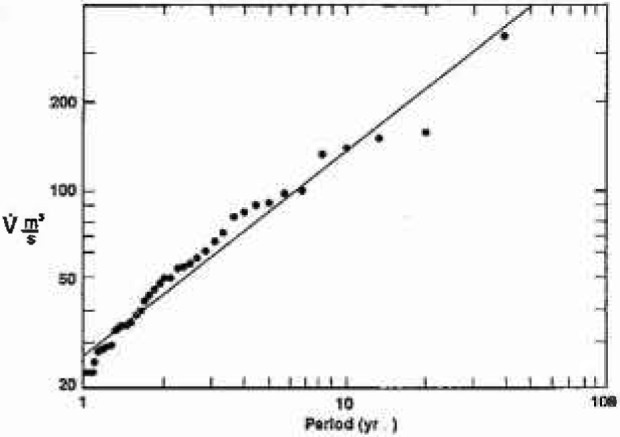
The peak daily discharge for the largest annual floods over 40 years as a function of the assigned period: Station 1-0860.

**Fig. 7b:**
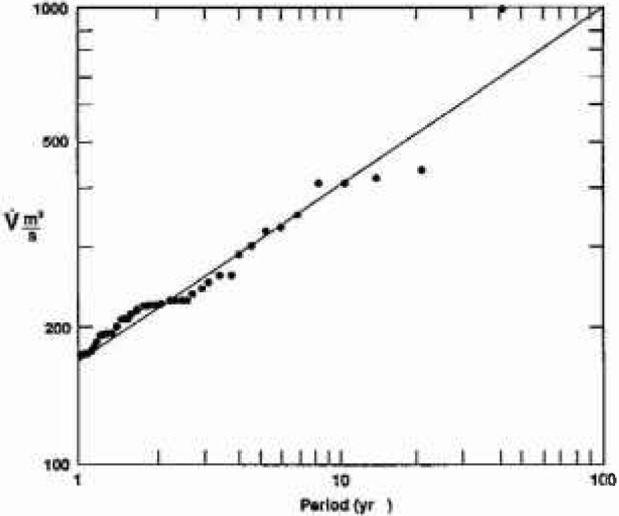
The peak daily discharge for the largest annual floods over 40 years as a function of the assigned period: Statton 3-2305.

**Fig. 8a:**
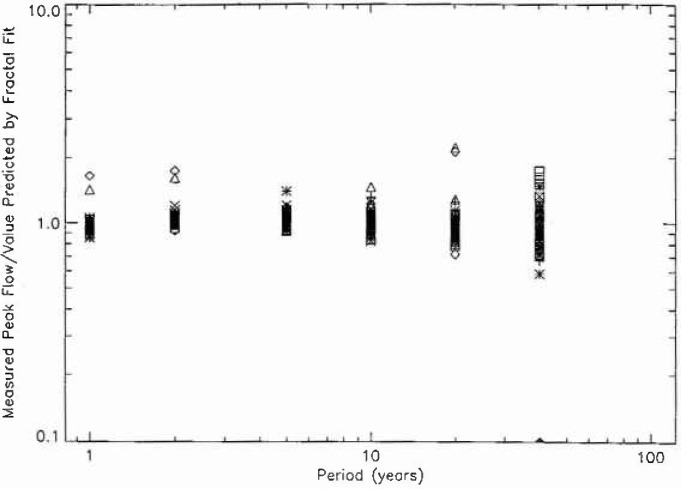
Ratio of the observed peak daily discharge to the value predicted by the fractal fit to the data as a function of the assigned period for the 111 stations in region 3.

**Fig. 8b:**
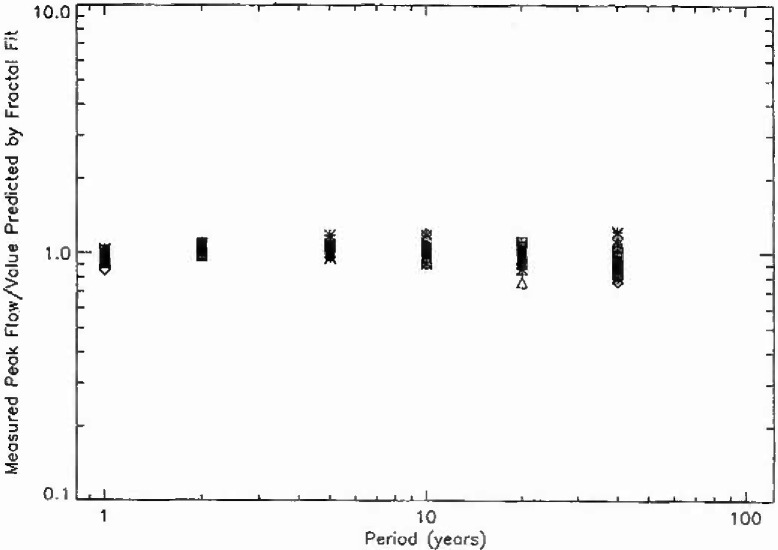
Ratio of the observed peak daily discharge to the value predicted by the fractal fit to the data as a function of the assigned period for the 57 stations in region 4.

**Fig. 8c:**
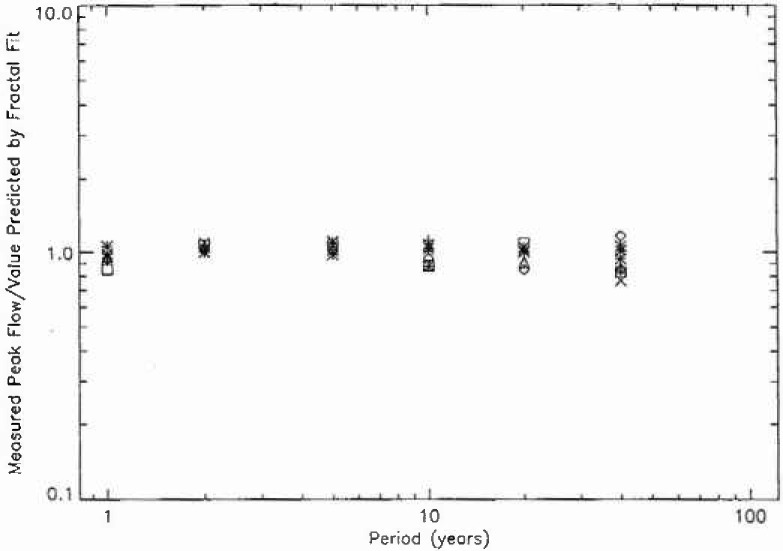
Ratio of the observed peak daily discharge to the value predicted by the fractal fit to the data as a function of the assigned period for the 10 stations in region 16.

**Fig. 8d:**
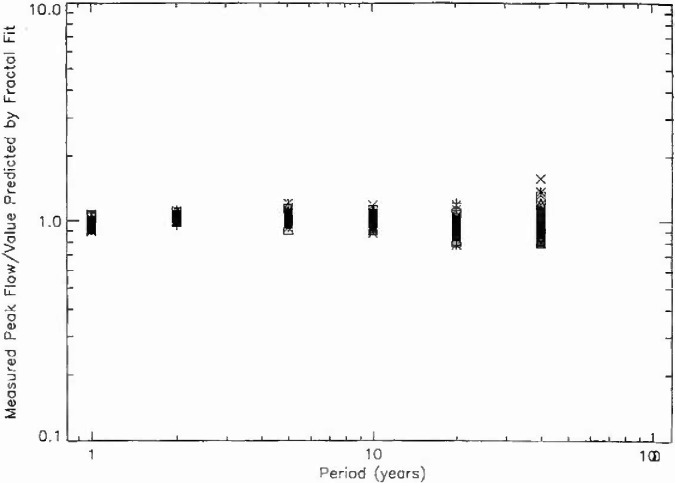
Ratio of the observed peak daily discharge to the value predicted by the fractal fit to the data as a function of the assigned period for the 100 stations in region 17.

**Fig. 9a:**
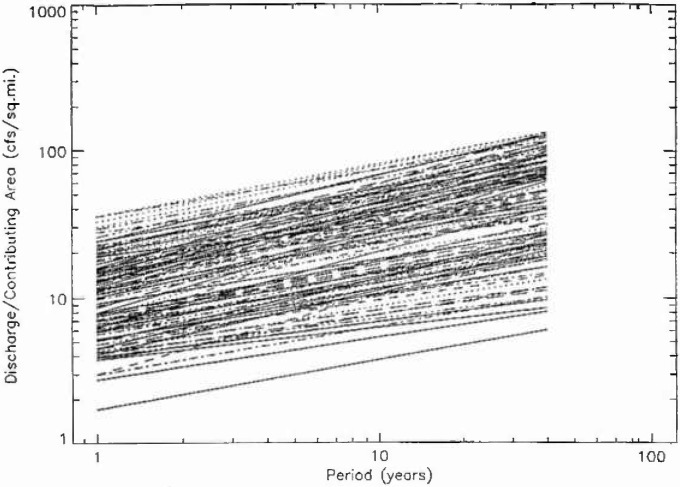
Fractal fits of the normalized flood frequency data for the 111 stations in region 3.

**Fig. 9b:**
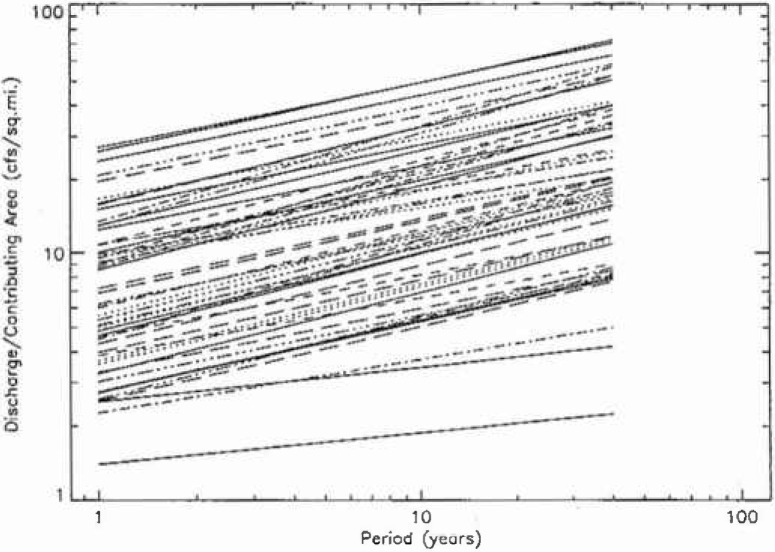
Fractal fits of the normalized flood frequency data for the 57 stations in region 4.

**Fig. 9c:**
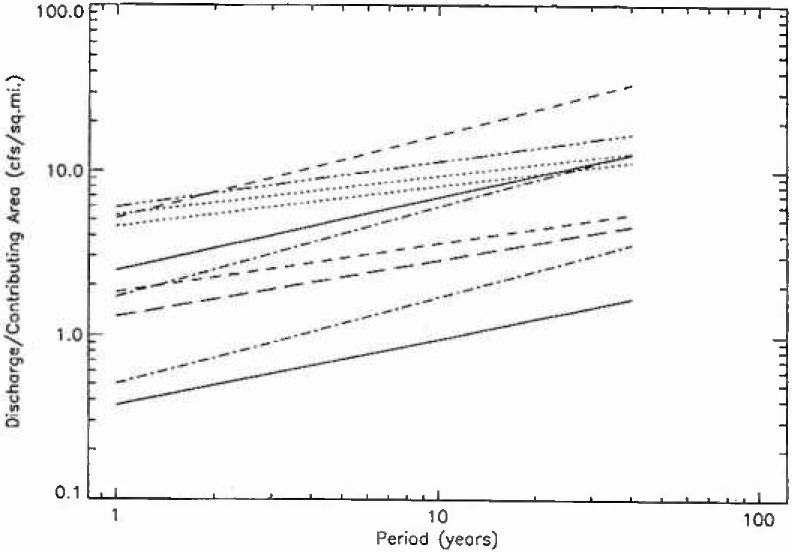
Fractal fits of the normalized flood frequency data for the 10 stations in region 16.

**Fig. 9d:**
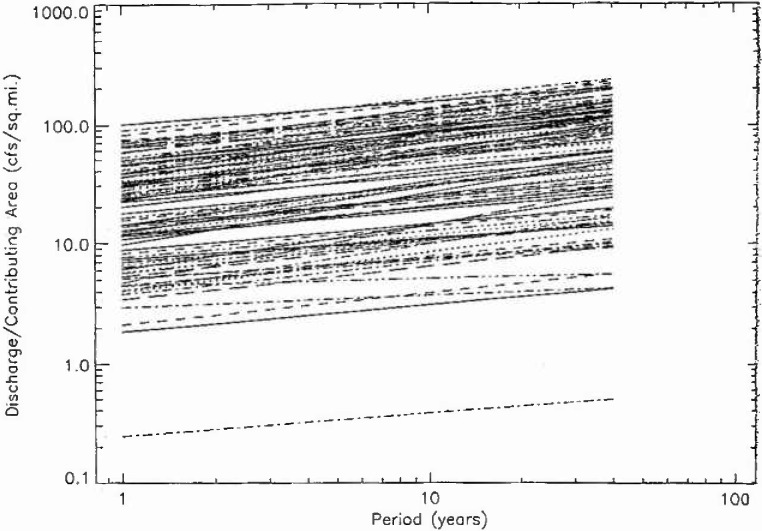
Fractal fits of the normalized flood frequency data for the 100 stations in region 17.

**Table 1:** Values of the Hausdorff measure *H*, fractal dimension *D*, flood intensity factor *F*, and Hurst exponent *H*_1_ for the 10 benchmark stations

Station	River	(State)	*H*	*D*	*F*	*H*_1_
1-1805	Westfield	(MA)	0.435	1.56	2.72	0.67
2-2185	Oconce	(GA)	0.540	1.46	3.47	0.72
5-3310	Mississippi	(MN)	0.470	1.53	2.95	0.72
6-3440	Little Missouri	(WY)	0.520	1.48	3.31	0.72
6-8005	Elkhorn	(NE)	0.540	1.46	3.47	0.67
7-2165	Mora	(NM)	0.630	1.37	4.27	0.73
8-1500	Liano	(TX)	0.719	1.28	5.24	0.70
10-3275	Humboldt	(NV)	0.616	1.38	4.13	0.66
11-0980	Arroyo Scco	(CA)	0.909	1.09	8.11	0.68
12-1570	Wenatchee	(WA)	0.310	1.69	2.04	0.72

**Table 2:** Average values and standard deviations of the flood intensity factor *F* for the 18 hydrologic districts

Hydrologic regions	*F*	Standard deviations	Number of stations
1	2.369	0.377	54
2	2.998	1.313	147
3	2.758	0.617	111
4	2.183	0.289	57
5	2.396	0.509	129
6	2.505	0.324	38
7	2.782	0.738	123
8	3.021	0.979	22
9	4.7	1.586	13
10	3.557	1.677	64
11	3.897	1.801	46
12	4.848	1.559	13
13	4.104	2.121	14
14	2.283	0.51	18
15	6.066	1.08	11
16	2.778	0.752	10
17	2.076	0.357	100
18	5.134	2.4	39
